# Evaluation of The Relationship of Circular RNA With Suicide Behavior In Patients Diagnosed With Schizophrenia and Other Psychotic Disorder

**DOI:** 10.1192/j.eurpsy.2024.227

**Published:** 2024-08-27

**Authors:** M. Aydin, S. O. Unal, E. M. Ozdemir

**Affiliations:** ^1^Psychiatry; ^2^Medical Genetics, Selcuk University, Konya, Türkiye

## Abstract

**Introduction:**

Schizophrenia is a major mental disorder with a high risk of suicide, which is one of the leading causes of early death in schizophrenia patients. It is known that suicidal behavior is 20-50 times higher in schizophrenia patients compared to the general population. Clinical features makes it difficult to determine the risk of suicide in this patient group. Since genetic studies on suicides of patients with schizophrenia are limited, this area was deemed worthy of research.

**Objectives:**

CircRNAs can potentially serve as minimally invasive biomarkers because they can freely cross the blood-brain barrier. It is aimed to define the effect of circRNA molecules on suicidal behavior in patients diagnosed with schizophrenia and other schizophrenia spectrum psychotic disorders, and to increase protective and preventive approaches by predicting possible consequences of suicidal behavior.

**Methods:**

104 patients followed up with the diagnosis of schizophrenia and and other schizophrenia spectrum psychotic disorders were included in the study. RNA was isolated from the blood taken into a hemogram tube, and three cirRNA molecules were identified using a number of RNA sequencing techniques. In addition, sociodemographic characteristics of the participants, clinical features of the disease, suicidal behavior history, current treatment status were questioned in detail. Simultaneously, the current clinical status was evaluated with clinical evaluation scales as Positive and Negative Syndrome Scale (PANSS), Calgary depression scale for schizophrenia (CDSS), Suicide Probability Scale (SPS), Beck Suicidal Intend Scale (BSIS).

**Results:**

Three circRNA molecules were identified, chr3_196488683, chr5_69175537 and hsa_circ_0084021. No significant difference was found between these molecules and past suicide attempts. It was found that chr5_69175537 was negatively associated with the age of onset of psychotic disorder negative symptoms, and hsa_circ_0084021 was negatively associated with the age of onset of both negative and positive symptoms. When the relationship between the clinical assessment scales and suicidal behavior was evaluated, the PANSS general symptoms subscale score was significantly higher in the group with suicidal behavior (p<0.05). CDSS mean scores and BSIS scores were also found to be significantly higher in the group with previous suicide attempts (p<0.01).

**Conclusions:**

Although our findings do not allow definitive conclusions due to the complex interaction between epidemiological and clinical factors and limited literature, it has shown that schizophrenia contains many risks that increase suicidal behavior. To predict suicide, circRNA molecules need to be supported by prospective studies with large sample groups and comparison with control groups.

**Disclosure of Interest:**

None Declared

